# A SWOT analysis of the complex interdependencies of the Maltese reimbursement processes

**DOI:** 10.1016/j.hpopen.2023.100095

**Published:** 2023-04-10

**Authors:** Katharina Abraham, Margreet Franken

**Affiliations:** Institute for Medical Technology Assessment, Erasmus University Rotterdam, Rotterdam, the Netherlands; Erasmus Centre for Health Economics Rotterdam (EsCHER), Erasmus University Rotterdam, the Netherlands; Erasmus School of Health Policy & Management, Erasmus University Rotterdam, Rotterdam, the Netherlands

## Abstract

**Background:**

The processes that operationalize the evaluation framework for new medicines are implemented to reach the system objectives of public health, financial sustainability, and equitability. However, when the activities and procedures of these processes are misaligned, the objectives of the system may be at risk.

**Objectives:**

To evaluate the supporting processes for introducing new medicines in public healthcare services in Malta.

**Methods:**

We first reviewed literature on the Maltese reimbursement system and subsequently conducted semi-structured interviews based on the Hutton Framework. Interviewees included policy makers, committee members, procurement staff, medical specialists, pharmacists, and pharmaceutical industry representatives. After validation, we analysed the data with a Strengths, Weaknesses, Opportunities, and Threats (SWOT) analysis.

**Results:**

Most medicines are assessed for introduction on the Government Formulary List. Exceptional requests fall outside this policy and pass through the Exceptional Medicinal Treatment route. Efficiency, quality, and transparency are major weaknesses across the supporting processes. Taking up responsibility, however, is considered the most important factor in reaching system objectives. Stakeholders tend to shift responsibilities to other processes, start/stop activities that impact the activities of subsequent processes whilst dismissing any contribution to the weaknesses of the system. Consequently, system objectives cannot be reached in an optimum manner.

**Conclusions:**

The Maltese case showed that recommendations for introducing new medicines in the public healthcare setting are influenced beyond the choice of HTA tools and criteria. Earmarked budgets, political steering, delays, and uninformed applicants as well as HTA capacity are impeding on system goals of public health, equity, and sustainability.

## Highlights

• Most drug reimbursement studies focus on the operationalization of tools and criteria.
• This study shows that procedures that support the system impact each other.
• Reimbursement systems can become more efficient by transforming weaknesses into strengths.
• Processes should be in alignment and functioning towards the reimbursement system’s goals.

## 1 Introduction

The promotion of public health stimulates health systems to strive for high-quality care at reasonable cost with health gains distributed equitably across the population [1], [2], [3], [4]. To achieve these objectives, countries established various approaches for newly introduced medicines. Germany and England promote health and sustainability by reimbursing all medicines with market approval unless included on a negative list [5]. In contrast, France, Italy, the Netherlands, and Sweden use a positive list including only medicines with reimbursement status to provide high-quality care and control budgets [4], [5]. Other mechanisms, such as disease- or population-group-specific schemes, cost sharing policies and managed entry schemes, further help to sustain systems financially, increase public health gains and distribute health equitably [6]. At the core, however, is the decision to fund a medicine commonly based on a systematic evaluation of the health technology’s value and budget impact. Health Technology Assessments (HTAs) are the main evaluation approach in most European countries [6]. Though the choice of HTA tools and criteria often differ, most countries established processes to support the operationalization of the evaluation framework [6], [7]. These processes work in alignment to reach the overall system objectives of sustainability, equity, and quality of care. Misalignment between processes, frictions and/or poor quality of outputs may impact the activities and procedures of other processes and eventually the objectives of the system. Current literature predominantly focuses on describing reimbursement systems (e.g., assessment and appraisal criteria, cost-effectiveness and budget thresholds) and evaluating methodologies (choice of comparator, study types, dealing with uncertainty) [4], [8], [9], [10], [11]. Our study adds to these aspects by unravelling interdependencies between the processes that facilitate HTAs based on the legislative and organizational set-up and the availability of HTA resources that allow for input and output exchange between stakeholders of those core processes, ultimately contributing to a better working of the reimbursement system. We selected Malta as case study as it is the smallest EU member state and, to our knowledge, Malta’s reimbursement system processes have not yet been evaluated. Malta is also one of the few EU countries where the Ministry for Health is the responsible authority for pricing and reimbursement as well as the public payer for in- and outpatient medicines [6]. Our aim was to evaluate the core processes of the Maltese system for introducing new medicines in public health services.

## 2 Methods

The evaluation of the core processes for introducing new medicines in the Maltese public healthcare services was based on a stepwise approach. First, we conducted a targeted literature review on the Maltese system (2008 to 2018) and subsequently carried out interviews. The aim of the interviews was to retrieve information on the set-up of the core processes and to discuss their advantages and disadvantages from the stakeholders’ view. The Hutton Framework [12] describing the policy implementation and technology decision level of reimbursement systems, together with its respective questionnaire was used to structure our interviews. We conducted two rounds of semi-structured face-to-face interviews. The first interview round included representatives from the Directorate for Pharmaceutical Affairs (DPA) (n = 10). In the second round, interviews were conducted with appraisal committee members (n = 10), medical (n = 3) and pharmacy representatives (n = 4), the pharmaceutical industry (n = 3), the procurement unit (n = 2), the hospital management (n = 1), and the Ministry for Health (n = 1). All interviews took place in Malta between December 2018 and April 2019, lasted 45 to 90 min, and were held in English language. Both authors were present at each interview, which were recorded when interviewees consented.

Based on the collected data, we drafted a description of the Maltese system and, after review by the interviewees and revision incorporating the feedback, evaluated the core processes of the system with a Strengths, Weaknesses, Opportunities and Threats (SWOT) analysis. SWOT analysis is a technique that helps identifying internal factors (strengths and weaknesses) and external factors (opportunities and threats) that influence a set of objectives [13], [14]. The SWOT analysis was revised after being shared with the interviewees for validation. Core processes were based on the legal set-up of the Maltese system and the established entities. In December 2022, we updated our findings after review and validation by two former interviewees who are representatives of the Ministry for Health in Malta.

## 3 Results

### 3.1 Government Formulary list

**Fig. 1:**
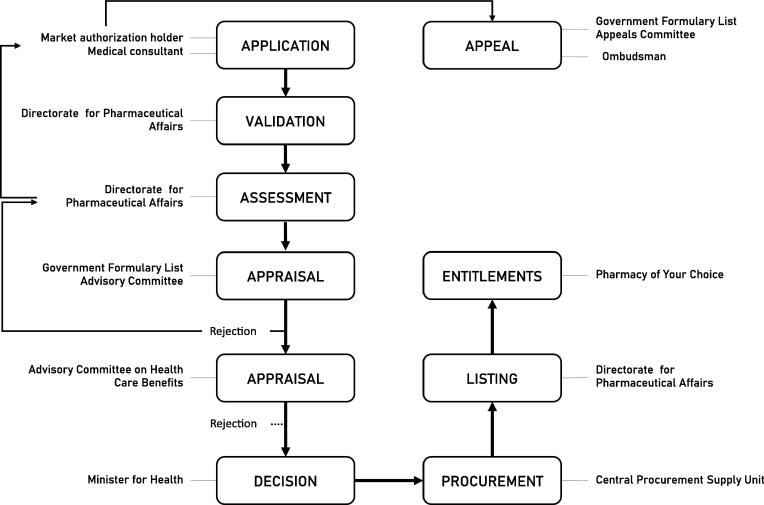
Core processes of introducing new medicines on the Government Formulary List.

In Malta, the financial coverage of medicines by the government is provided to any person suffering from a chronic health condition listed on Schedule V for free at point of delivery [15]. Most new medicines pass through the process of introduction on the Government Formula List (GFL) route. Market authorization holders (MAH) and local medical specialists can submit applications. All eligible medicines are assessed by DPA’s HTA Unit that drafts HTA reports for the Government Formulary List Advisory Committee (GFLAC). The GFLAC appraises the benefits and costs of the new medicine compared to other medicines on the same clinical pathway. GFLAC’s recommendation can be appealed by the applicant. GFLAC advises the Advisory Committee on Health Care Benefits (ACHCB). The ACHCB appraises the medicine based on affordability (i.e., budget impact), sustainability and capacity of the system. The Minister for Health endorses the recommended medicines, which are then purchased by the Central Procurement Supply Unit (CPSU) through public tenders or negotiations. Once procured, DPA’s Formulary Management Unit (FMU) updates the public GFL documents and informs relevant stakeholders through circular drafts issued by the CMO’s (Chief Medical Officer) Office. In- and outpatient pharmacies can request new medicines from CPSU and the central Pharmacy of Your Choice (POYC), respectively. POYC further issues the entitlements providing patients with access to new medicines at their local POYC. Fig. 1 presents the core processes of the GFL route.

#### 3.1.1 Application and validation

The set-up of the application and validation process contributes to the objectives of the Maltese system in two major ways. Firstly, it supports the submission of potentially beneficial medicines targeting chronic health conditions and, secondly, gatekeeps the system’s sustainability through eligibility criteria. Its main strengths include accessibility (documents are online available), no application fees, and support with finalizing submissions. The use of Standard Operating Procedures (SOPs) and forms facilitate the validation process and ensure new medicines target conditions eligible for public funding. Thereby, effectively gatekeeping the sustainability of the system. The SOPs include a time cap of five working days to validate submissions and to inform applicants on the status, thereby, strengthening the relationship with industry. The system further aims to foster and steer submissions of new medicines for certain prioritised conditions as publicly announced by the Minister of Finance in the national budget speech. Political steering and financial constraints may alter these, resulting in tension with industry and lower submission rates for prioritized and non-prioritized disease areas. Similar negative effects on submission rates can be expected due to the long assessment and appraisal processes regularly violating the EU Transparency Directive of 180 days applicable to submissions from MAH [16]. The application and validation process might benefit from horizon scanning to promote the submission of applications for prioritized disease areas. Furthermore, horizon scanning can potentially inform the prioritization list.

#### 3.1.2 Assessment

The set-up of the assessment process supports decision-makers with evidence-based information to inform trade-offs between public health gains, costs, and budget impact. A main strength is the collaboration with local medical experts on drafting clinical pathways as data from literature and other HTA agencies may not be transferable to the Maltese setting. Considering that all validated submissions need to be assessed, requiring one to two months, assessors are assigned to the same disease areas for efficiency. Medicines for prioritized disease areas are assessed first. When prioritization changes, HTA reports may become irrelevant, and medicines are backlogged. When put back on the agenda, the reports often need revision. For prioritized medicines, reports need to be drafted quickly, which may impact the quality of the assessment. Another weakness is the lack of a procedure to track market approvals for medicines relevant to the pathway(s). Consequently, (new) medicines are not included in the assessments and appraisals. Horizon scanning may be an opportunity to include new medicines earlier in the assessments. The lack of access to scientific journals limits the systematic evaluation of evidence. Evidence from pragmatic literature review is commonly opposed by medical experts during appraisal. Accessible and reliable data remains a weakness also for budget impact (BI) calculations. The pricing for new medicines depends, amongst others, on the list prices of 12 countries. Not all new medicines have these prices readily available, which limits the calculation of reference prices. Volumes are primarily based on input from local medical experts and may vary across experts or change after approval. Incorrect price and volume estimates can result in budgetary issues (e.g., higher purchasing prices) and/or under- or overstock of medicines impacting public health and sustainability goals. Improvements could be achieved through a membership at the European medicine price database (EURIPID). Assessors provide cost-effectiveness information from the company submission and international HTA agencies but lack expertise to comprehend cost-effectiveness evidence. Moreover, transferability issues reduce the use of cost-effectiveness information from other countries. This limits decision-makers in assigning monetary value to the additional health gain of a new medicine in a transparent manner and to efficiently allocate budget. This weakness is addressed as the Maltese Ministry for Health received funding for health economic training from the European Structural and Investment Fund.

#### 3.1.3 Appraisal

The set-up of the appraisal process including two committees contributes to sustainability and public health through recommendation of effective and cost-effective medicines that fit the yearly budgets. This process shows, however, several weaknesses, such as the lack of explicit appraisal criteria and budget threshold, the limited role of cost-effectiveness, and delay of appraisals. In GFLAC’s appraisals, criteria are not explicitly stated but committee members consider the extension of life most important. Thresholds are subject to internal discussions. The lack of explicit criteria and thresholds reduces transparency and consistency in decision-making possibly impacting public health goals. Stakeholders also questioned whether GFLAC should appraise BI, since the second committee, the ACHCB, considers BI within its remit. The ACHCB also lacks explicit criteria but focuses on keeping track of all health expenditures for the subsequent three years to achieve financial sustainability. The lack of sound BI calculations reduces the quality of BI appraisals and may, therefore, threaten the financial sustainability of the system. The role of cost-effectiveness is challenged by both appraisal committees because of transferability issues. More importantly, health economic skills are limited to properly appraise cost-effectiveness evidence limiting possible gains in sustainability and public health. Cost-effectiveness skills, currently acquired by the assessors pose an opportunity to utilize cost-effectiveness data in the appraisals and to transfer learnings to the committees. The implementation of a cost-effectiveness criterion and budget threshold at ACHCB level would enable informed decision-making regarding maximum public health gains, which can be openly communicated to stakeholders. Delays in appraising new medicines is a shared weakness of GFLAC and ACHCB. The planned monthly appraisal meetings by GFLAC were not attained in any of the years between 2014 and 2019, ranging between 3 and 9 meetings per year. The ACHCB on average convenes three times per year only (2014 to 2019). Changes in prioritization of disease areas and irregular appraisal meetings can delay decision-making at both committees resulting in changes on the agenda and/or a backlog of sometimes several years. Thereby, largely impacting the system’s public health objective. Between 2014 and 2019, up to 35% of appraised medicines were backlogged by GFLAC (Table 1). At ACHCB level between 5% and 50% of treatments were set to pending per year (Table 2).

**Table 1:** Outcomes of treatments appraised, per year – Government Formulary List Advisory Committee (GFLAC), 2014–2019.

Year	Number of treatments	Approved	%	Rejected	%	Pending	%
2014	**100**	73	73%	22	22%	5	5%
2015	**40**	17	43%	9	23%	14	35%
2016	**33**	26	79%	6	18%	1	3%
2017	**17**	11	65%	5	29%	1	6%
2018	**39**	24	62%	11	28%	4	10%
2019	**30**	29	97%	1	3%	0	0%

**Table 2:** Outcomes of treatments appraised, per year – Advisory Committee for Health Care Benefits (ACHCB), 2014–2019.

Year	Number of treatments	Approved			Rejected			Pending		
		Total	*	%	Total	*	%	Total	*	%
2014	**10**	5	*–*	50%	1	*–*	10%	4	*1*	40%
2015	**54**	42	*4*	78%	6	*–*	11%	6	*3*	11%
2016	**4**	2	*2*	50%	0	*–*	0%	2	*1*	50%
2017	**21**	16	*12*	76%	4	*3*	19%	1	*–*	5%
2018	**30**	22	11	73%	5	4	17%	3	2	10%
2019	**12**	8	5	67%	0	0	0%	4	0	33%

*Of which oncology treatments.

#### 3.1.4 Decision

The Minister for Health is accountable for achieving the system’s objectives by supporting the processes, endorsing legislation, appointing committee members, and allocating budgets. The stipulation of the remits of the appraisal committees is seen as weakness that creates inefficiencies and frictions between the two committees. Additionally, the current set-up of the processes does not contribute to the EU Transparency Directive of 180 days, nor are procedures in place to support the Directive resulting in delays and, thus, limited access to medicines. The composition of GFLAC lacks health economics expertise. The appointment of a health economist is seen as opportunity to contribute to sustainability and public health objectives. Although, the composition of ACHCB represents a wider range of expertise, amongst others economic expertise (but no health economic expertise), members have competing responsibilities resulting in no-shows and delays in appraisal. The Minister allocates budgets to specific disease areas that would otherwise fall under the general budget (e.g., oncology). This reduces the budget for other medicines possibly resulting in displacement of healthcare, especially when earmarking of budgets is politically driven rather than evidence-based. Stakeholders outside the system emphasized the lack of innovative treatments, which are readily available in other countries. Thus, budgets for new medicines might be generally too low to cover rapidly advancing new and innovative medicines. The uptake of cost-effectiveness as criterion at assessment and appraisal would enhance efficient use of available budgets. Alternatively, although politically sensitive, budgetary constraints could be eased through the introduction of co-payments.

#### 3.1.5 Implementation

The implementation processes are crucial for reaching public health and sustainability objectives. The procurement of new medicines, their listing on the GFL and issuing of entitlements provide patients with access to new medicines whilst controlling expenses. Strengths of the procurement process are the application of SOPs for issuing tenders and the clear set of procurement criteria (e.g., lowest price). An external committee reviews the public tenders and supplier selection ensuring fair competition and compliance with pricing criteria. The lack of reliable epidemiological and pricing data leads to inefficiencies on procurement side due to limited negotiation power and under- or overstocking threatening medicine availability and financial sustainability. A surge in appeals further reduces effective procurement resulting in delays in treatment availability. Industry resolved to appeal tenders on grounds of unfair competition at procurement level after rejection letters for GFLAC recommendations were temporarily halted and, therefore, applicants were not informed on the outcome of their submission. Appeals are also issued for medicines relevant within the clinical pathway but not yet appraised when market approval was issued after HTA reports were drafted. Consequently, tenders were issued with open specifications to allow for competition. This approach though resulted in dissatisfaction amongst medical experts and patients who request specific medicines. Moreover, when approved medicines are successfully purchased, this is often not communicated to the subsequent processes. Stakeholders, such as medical specialists (i.e., consultants) and pharmacies, remain uninformed resulting in further delays. The circular drafts issued to inform stakeholders might not overlap with re-stocking schedules of inpatient pharmacies. Access is further complicated by the logistic arrangement of stocks. A new system where suppliers directly deliver pharmacies might ease storage issues. Table 3 provides the main findings of the SWOT analysis; supplementary Table S1 provides the details for each of the GFL core processes.

**Table 3:** Main findings of the SWOT Government Formulary List across Core Processes*.

**Strengths**	▪ Application procedure is accessible; no application fee (V&A, A). ▪ Validation SOPs are utilized ensuring fair and timely processing (V&A). ▪ Public yearly prioritization list promoting and steering applications (D, V&A, A). ▪ Collaboration with medical consultants to draft setting-relevant clinical pathways (A). ▪ Efficient assessment procedure (i.e., assessors are assigned to the same conditions) (A). ▪ Clear purchasing criteria for tenders (I). ▪ A separate committee reviews public tenders and selection reason to ensure fair competition and compliance with pricing criteria (I).
**Weaknesses**	▪ Discrepancy between prioritization list and internal yearly medicine/disease priorities (A&V, A, AP). ▪ EU Transparency Directive of 180 days is regularly violated (A&V, A, AP, I, D). ▪ Limited access to scientific journals (A, AP). ▪ Limited/Lack of health economic expertise (A, AP). ▪ Limited use of cost-effectiveness information (A, AP, D). ▪ Lack of national epidemiological and pricing data to calculate minimum reference price and estimate budget impact (A, AP, I). ▪ Appraisal criteria are not clearly defined (AP, A&V). ▪ Overlapping committee remits (AP, D). ▪ Unregular appraisal meetings (A, AP). ▪ Competing responsibilities of appraisal members (AP). ▪ Lack of operationalized budget threshold. ▪ Insufficient funding of new & innovative medicines within healthcare budget (AP, I, A&V). ▪ Lack of evidence-based earmarked budgets (D). ▪ New medicines are not considered in the HTA reports and/or rejection letters are not sent to the applicant (A, I). ▪ Procurement of formulations different than approved (i.e., open specifications) (I, A). ▪ Lack of communication of the procurement outputs as input for other processes (e.g., actual patient numbers and prices for following clinical pathway including that medicine). ▪ Lack of communication between successful procurement of approved medicines and inpatient pharmacies who have set re-stocking cycles.
**Opportunities**	▪ Horizon scanning (A&V, A). ▪ Membership with EURIPID (A). ▪ Funding from the European Structural and Investment Fund for health economics training (A, AP). ▪ Legitimizing cost-effectiveness as evaluation criterion within the required economic evaluation stipulated by legislation (A, AP). ▪ Co-payments (D). ▪ Supply chain innovations (I).
**Threats**	▪ Approval of new medicines by EMA or the local market authorization does not align with assessment procedures (A&V, A, AP, I). ▪ Transferability of cost-effectiveness, cost, and effectiveness data from other countries to the Maltese healthcare setting (A, AP, I). ▪ Reference prices provided by basket countries are not available for all medicines (A, AP, I).

A = Assessment; AP = Appraisal; A&V = Application & Validation; D = Decision; EMA = European Medicine Agency; EU = European Union; HTA = Health Technology Assessment; I = Implementation; SOP = Standard Operating Procedures.

* In brackets all impacted processes.

### 3.2 Exceptional Medicinal Treatment

**Fig. 2:**
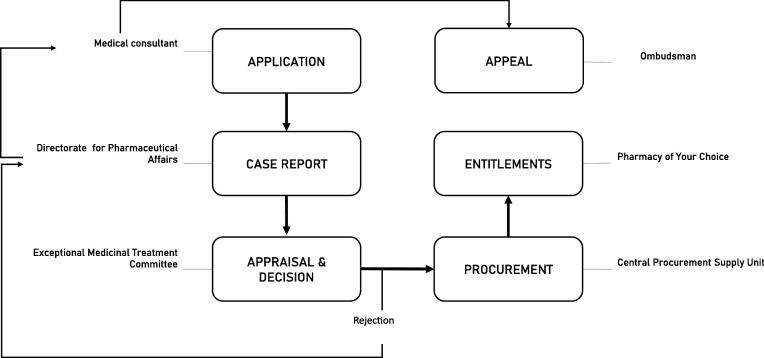
Core processes of introducing new Exceptional Medicinal Treatments.

The Exceptional Medicinal Treatment (EMT) route ensures treatment for individuals with exceptional medicinal needs not covered by the GFL or associated policies. EMT requests are submitted by medical specialists. For requests with an indication never previously assessed, the EMT Unit within DPA compiles a short case report on efficacy and costs of the medicine. The EMT Committee (EMTC) appraises and decides on the approval. Exceptionality is the main appraisal criterion. Approved medicines are purchased by CPSU usually through confidential negotiations. The procured medicine is directly forwarded to the inpatient pharmacy or, in case of an outpatient medicine, to POYC who issues the entitlement and forwards the medicine to the patient’s local pharmacy. Fig. 2 presents the core processes of the EMT route.

The processes surrounding EMTs support the objectives of public health, sustainability, and especially equity. A main strength is only new EMT requests require assessment, thereby, increasing efficiency, which is further promoted by keeping assessment time down to two to three weeks, and in case of urgent matters to one day. Although the recent shortening of the application form facilitates medical specialists, it provides less information for the assessment, which must be then collected by the assessor. The EMT committee meets every three weeks to appraise the requests based on a list of criteria with ‘exceptionality’ and ‘listing on the GFL’ being the main criteria. The internally handled definition of exceptionality (max. 10 patients) is, however, unknown outside the committee resulting in requests belonging on the GFL. The submission of ineligible requests takes time from processing eligible EMT requests reducing efficiency. Decisions on funding are communicated without much delay contributing to a trusting relationship with the applicants. Historically, the efficient processing combined with the ambiguous exceptionality criterion, high approval rates and faster procurement stimulated applicants to submit applications as EMT requests. Several interviewees stated that this historic overuse still poses a threat to the financial sustainability especially since previously approved EMTs are generally renewed. Budgetary pressure may further increase when patient numbers significantly change after approval. Since CPSU operates on economies of scales, negotiation power is lost, and additional medicines need to be purchased at higher cost. Budgetary responsibility for EMTs is viewed differently by stakeholders. Some insisted the budget was not available to the committee whereas others assured the budget was held by the CMO. Legislation stipulates the CMO, or a representative of the CMO, as EMTC member, thus budget may or may not reside within EMTC. Lacking budget information and responsibility may impact sustainability of the system. In addition, the small population of Malta may result in frequent EMT needs placing further pressure on EMT budgets. An opportunity to promote financial sustainability and public health may be the use of cost-effectiveness evidence. Operating a EMT-specific cost-effectiveness threshold may improve efficient allocation of budgets in an equitable manner, for example, by assigning higher thresholds for severe diseases. The use of cost-effectiveness evidence can also be a tool for balancing equitable and equal access. Table 4 provides the main findings of the SWOT analysis; supplementary Table S2 provides the details for each of the EMT core processes.

**Table 4:** Main findings of the SWOT Exceptional Medicinal Treatments across Core Processes*.

**Strengths**	▪ A new policy distinguishing three application requests (new requests, renewals, and requests for previously approved indications) reduces bureaucracy tendering to medical consultants (AP, A, A&D). ▪ Short request form (1 page) caters to applicants (AP). ▪ Regular appraisal meetings of the EMTC (every 2–3 weeks) facilitating faster access of approved medicines (A&D, I).
**Weaknesses**	▪ The short request form reduces the evidence input by clinicians which then needs to be collected by the assessors (AP, A). ▪ Historically, the EMT route provided a possibility to access medicines faster than when applying on the GFL. Although nowadays, requests are assessed more critically, previously approved medicines remain within the EMT route; requests are still renewed and procured (A&D, I). ▪ Unclear operationalisation of reimbursement criterion ‘exceptionality’ (AP, A, A&D, I). ▪ Unclarity around budget and budgetary responsibility by EMTC; some stakeholders claimed no separate budget was available, others claimed budget was available and under the responsibility of the CMO (A&D, I).
**Opportunities**	▪ Application of CE criterion as an opportunity for sustainability (A, A&D). ▪ Application of CE criterion and threshold as a tool for balancing equitable and equal access to medicines (A, A&D). ▪ Threshold suitable for orphan medicines to provide budget indication (A, A&D).
**Threats**	▪ The very purpose of the EMT route limits procurement in volumes and therefore, impacts financial sustainability of the system (I). ▪ The small population of Malta may have more frequently EMT needs relative to countries with larger populations (AP, A, I).

A = Assessment; A&D = Appraisal & Decision; AP = Application; CE = Cost-Effectiveness; CMO = Chief Medical Officer; D = Decision; EMT = Exceptional Medicinal Treatments; EMTC = Exceptional Medicinal Treatment Committee; I = Implementation.

* In brackets all impacted processes.

## 4 Discussion

We evaluated the core processes of the Maltese system introducing new medicines in public healthcare services. Our findings show that activities at one level of the system impact activities of other processes, often to a large extent. System’s strengths contribute to reaching system goals and weaknesses negatively impact activities across processes, thereby threatening the system goals of public health, equity, and sustainability.

One of the main weaknesses challenging the public health objective is the delay across all processes. The EU Transparency Directive stipulates a maximum of 180 days [16]. Interviewees stated that this is commonly exceeded with some medicines being processed years after submission. In case of submissions from clinicians no official timeframe applies. Most Central and Eastern European Countries stay within the 180 days through simplified procedures with shorter timelines and clock-stop mechanisms [17], [18]. Another determinator is the type of assessment. A EUnetHTA report showed that Multiple Technology Assessments (MTAs) and re-assessments commonly led to longer timeframes exceeding 200 days [19]. Although Malta mainly conducts MTAs, there are other factors that play a role in the delays. Our study shows that changes in disease prioritization impacts activities across processes. Industry needs to prepare new submissions, assessments render outdated, and appraisals of non-prioritized medicines are put to halt. Changes in prioritization are mainly due to political steering as clear priority-setting processes that include selection methods and participation of stakeholders are lacking in Malta. Novaes and de Soarez [20] found that many countries lack (clear) priority-setting criteria, and only half of their assessed countries had a panel or committee to arrive at recommendations for prioritization. The HTA agencies in the UK and Canada had the most criteria for priority setting including budget impact, burden of disease, medical impact of the technology and the interest of governments, health practitioners and patients [20]. Improvements in prioritizations could be achieved using horizon scanning by providing timely, early information on potential new medicines [22].

Funding decisions in Malta are further delayed by irregular appraisal meetings for medicines with no earmarked budget. Ades et al. [21] found that decisions are less likely to be delayed when budgets are available. To increase the budget, Malta could introduce co-payments, although political sensitive. Studies have shown that (introducing) co-payments may reduce public pharmaceutical expenditure [6]. However, they also showed that eliminating co-payments improved medication adherence among lower economic residents. Therefore, implementing co-payments would be a trade-off between health system goals, especially for specific populations, thereby threatening equity objectives.

The lack of communication on application status and decision contradicts the EU Transparency Directive. In Malta, applicants remain unaware, sometimes for several years, about the funding decision and whether efficacy, costs, cost-effectiveness, or budget impact resulted in the rejection of the medicine. Whilst the justification of decisions may be communicated to applicants in other countries, they are rarely made publicly available [23]. An exception might be the Netherlands, where appraisal meetings are open to the public albeit not all requests are discussed in the appraisal meeting [7]. In the Maltese system, the set of criteria and their relative weight are not formulated transparently nor are assessment and/or appraisal reports available to the public or communicated to the applicant.

The lack of reliable input data and robustly calculated economic outcomes showed to be a main weakness of the Maltese assessment process with large implications to subsequent processes and overall system objectives. For budget impact calculations assessors rely on expert opinion in absence of registries. Misalignment of medical experts emphasize uncertainties and question reliability. Lacking access to a pricing database and limited health economics expertise further reduces the robustness of HTAs. Although a membership with EURIPID pricing database would be possible, it requires sharing of Malta’s own pricing data, which could be hampered by stakeholders of other processes to keep negotiation power during procurement. Sufficient resources and HTA capacities are needed for a well-functioning HTA system [25]. A shortage of health economic capacities and resources was found in several countries especially in Eastern Europe [8], [23]. Czech Republic, Hungary, Romania, Bulgaria, and Poland all reported a shortage in trained professionals. Bulgaria, therefore, outsourced assessments to external experts [8]. To build local HTA capacity, investments are needed in the educational system for HTA graduate and postgraduate programs [24]. The Maltese system may benefit from similar investments in local capacity. It should, however, be noted that the Maltese Ministry for Health received funding for health economic training from the European Structural and Investment Fund. DPA assessors are currently acquiring knowledge on using cost-effectiveness data in their assessment reports [22]. Meanwhile, outsourcing the health economic assessment to external experts might be beneficial.

The market size of Malta could be considered a threat to the submission of new and innovative medicines as well as to the sustainable procurement of medicines. However, this was not confirmed by stakeholders who considered the Maltese market as desirable for the pharmaceutical industry. Although earmarked budgets were considered a strength by most stakeholders, there is a potential threat to the system objectives as other public health may be displaced, which extend has not been evaluated, for example with cost-effectiveness tools.

Whilst efficiency, transparency, and quality showed to be the main weaknesses of the system processes, taking responsibility is probably the most important factor in reaching system objectives. Our study showed that stakeholders within the system tend to shift responsibilities to other processes. Activities that negatively impact other processes may be started or stopped (e.g., information on application/procurement status) whilst dismissing their own contribution to system weaknesses (e.g., delays). These interdependencies have been little discussed in literature. Whilst the choice of HTA tools and criteria and their implementation and transparency are key aspects in decision-making, the technical and ethical aspects may fail to consider the activities of processes impacting each other. Allen et al., for example, found that differences in reimbursement recommendations were due to different handling of uncertainties and comparator choice [9]. The Maltese case, however, shows that recommendations are very much influenced by earmarked budgets, political steering, delays, the decision not to inform applicants as well as HTA capacity, all impacting decision-making and access to new treatments through misalignment of supporting processes.

It should be noted that many of the identified weaknesses have currently been tackled by DPA. In 2020, DPA introduced a horizon scanning system to improve strategic financial planning and decision-making [25] and DPA aims to further expand this to inform prioritisation setting. Moreover, DPA introduced regular meetings with clinicians before the GFLAC meetings to align and better support GFLAC in their appraisal. Furthermore, the surge in appeals was reduced through better planning of HTAs, internal Horizon Scanning, and meetings with MAHs. DPA has also resorted to HTAs by class approach, when possible, to facilitate competition via open specifications. The no-show of ACHCB members and resulting backlog of medicines was solved after a change in the leading positions at the Ministry for Health (see supplementary Tab. S3-S5). Other improvements include the re-opening of the discussions on membership with EURIPID, the development of a standardised pharmacotherapeutic and pharmacoeconomic assessment framework, training in health economics and the conduction of two shadow HTA’s with two Dutch institutes.

Some of the external factors that were originally identified as opportunities have changed over the past years. For many years, medicine supplies were mainly derived from the UK based on established relationships with wholesalers which was disrupted due to the Brexit. Consequently, the market size of Malta can now be seen as a threat to system objectives. Similarly, the anticipated growth in GDP will most likely be hampered due to the COVID-19 pandemic and the onset of the conflict between Russia and Ukraine.

We believe that our findings are based on data thoroughly collected, analyzed, and validated by different stakeholders and represent a variety of views across the Maltese system. Although the number of interviewees per process could have been more balanced, we do not anticipate an impact on our findings. Saturation was reached as most findings were shared across stakeholders. Nevertheless, even though invited for an interview, the views of the Permanent Secretary and CMO are missing, which could have enriched our findings. Although since the conduction of interviews some SWOT may no longer apply, the most important recent changes were, however, included and validated with previous interviewees within the Ministry for Health. We believe that our findings are of value for other countries who may consider to carefully assess their own processes and interdependencies that can frustrate and delay reimbursement processes in order to improve alignment and cooperation between stakeholders and, thus, their system objectives.

To conclude, this study provides detailed insights in the interdependencies between processes and their potential for impacting system objectives. Although systems differ across countries, our findings on the weaknesses of processes, namely the lack of efficiency, transparency, quality, and responsibility can be generalized to various jurisdictions. Countries, especially those with a shorter HTA history, limited resources and expertise could optimize their reimbursement systems by reviewing their core processes considering the SWOT found for Malta. By ensuring transforming weaknesses into strengths, systems might become more efficient and effective in achieving public health, equity, and sustainability.

## CRediT authorship contribution statement

**Katharina Abraham:** Conceptualization, Methodology, Investigation, Data curation, Visualization, Validation, Writing – original draft. **Margreet Franken:** Conceptualization, Methodology, Investigation, Validation, Writing – review & editing, Supervision, Project administration, Funding acquisition.

## Declaration of Competing Interest

The authors declare that they have no known competing financial interests or personal relationships that could have appeared to influence the work reported in this paper.
