# Supplementary material for: A SWOT analysis of the complex interdependencies of the Maltese reimbursement processes
Source: Health Policy Open. 2023 Apr 10;4:100095. doi: 10.1016/j.hpopen.2023.100095 (PMC10297753; doi:10.1016/j.hpopen.2023.100095)
Supplement: Supplementary data 1 [file mmc1.pdf]

Supplementary materials

Supplementary Table S1 - SWOT Government Formulary List Core Processes

	**Application & Validation**
**Strengths**	▪ Application procedure is accessible; no application fee<br>▪ Validation SOPs are utilized ensuring fair and timely processing<br>▪ Public yearly prioritization list promoting and steering applications (Strength, introduced by other processes of the system)<br>▪ Support to applicants in finalizing submissions
**Weaknesses**	▪ No checks are in place for applicants applying wrongly on the GFL with medicines that belong on the EMT route resulting in procurement of those medicines on GFL basis and, thus, GFL budget rather than EMT budget<br>▪ Discrepancy between prioritization list and internal yearly medicine/disease priorities may result in lower submission rates (Weakness, introduced by other processes of the system)<br>▪ EU Transparency Directive of 180 days is regularly violated may result in lower submission rates (Weakness, introduced by other processes of the system)
**Opportunities**	▪ Growth in GDP resulting in larger budgets for new medicines<br>▪ EU membership enhances chances of receiving innovative treatment applications<br>▪ MAHs desire to submit applications<br>▪ Horizon scanning to promote and steer applications

**Threats**	▪ Application for treatments that require specific tests (e.g., genetic tests) unavailable in Malta<br>▪ Market approvels by the UK authority for the Maltese setting are no longer possible after Brexit potentially reducing submission rates
	**Assessment**
**Strengths**	▪ Collaboration with medical consultants to draft setting-relevant clinical pathways<br>▪ Assessors have strong expertise on pharmacological aspects of new medicines<br>▪ Efficient assessment procedure (i.e., assessors are assigned to the same conditions)
**Weaknesses**	▪ Assessors have limited access to scientific journals<br>▪ Assessors have limited health economic expertise<br>▪ Assessors make limited use of CE information<br>▪ BI calculations based on patient numbers provided by clinical experts have been considered as not representative by other medical experts and procurement<br>▪ Lack of pharmacoeconomical robustness in the MRP calculation resulting in unreliable prices (e.g., skewed prices due to lacking prices in the basket)<br>▪ No standard format of HTA reports and no internal reviews resulting in quality and structural differences across assessors

	▪ Lack of collaboration between assessors hampering improvements in the quality of assessments<br>▪ Lack of guidance by assessors on the additional benefit of assessed medicines is considered a challenge by the appraisal committee (GFLAC)<br>▪ Political direction within the MfH to prioritize some medicines over others may result in outdated HTA reports, delayed assessments, and inefficiencies<br>▪ Lack of staff to handle the workload of additional submissions triggered by the publicly announced prioritization list
**Opportunities**	▪ EUnetHTA participation enabling collaboration on HTAs with other countries<br>▪ EU HTA legislation strengthening cross-country collaboration on HTAs<br>▪ Utilization of novel medicine assessments published by other HTA agencies<br>▪ Membership with EURIPID to access larger pricing datasets<br>▪ Funding from the European Structural and Investment Fund for training on health economics<br>▪ Collaboration of medical experts on treatment pathways and protocols can result in more accurate input data and alignment on assessments<br>▪ Horizon scanning as part of the joint work done within EUnetHTA to help identify emerging health technologies

**Threats**	▪ Potential discontinuation of EUnetHTA funding after 2021 and consequently the production of relative effectiveness assessment reports<br>▪ Transferability of CE, cost and effectiveness data from other countries to the Maltese healthcare setting<br>▪ European Transparency Directive of 180 days to finalize the evaluation of new medicines<br>▪ Approval of new medicines by EMA or the local market authorization does not align with assessment procedures<br>▪ Community funds and other committees with the ability to provide funds for medicines interfere with the system's prioritization assessing new medicines (e.g., The Community Chest Fund and the Committee for abroad treatment, both provide treatment to patients without assessing the evidence who then must apply at the system for continuation of the treatment)<br>▪ Lack of legitimisation of the system by stakeholders outside the system due to lacking awareness and understanding of the processes and rationales for decision-making<br>▪ Lack of databases on population health (e.g., diseases/conditions and patient numbers)<br>▪ High immigration rates change the country's epidemiology requiring different data inputs for the assessments<br>▪ Reference prices provided by basket countries are not available for all medicines

	Appraisal (GFLAC)
**Strengths**	▪ Committee member representing the public's wishes and concerns
**Weaknesses**	▪ Committee members with limited health economic expertise to appraise BI and CE<br>▪ The range and relative weight of the appraisal criteria are not clearly defined resulting in untransparent recommendations<br>▪ Discrepancy between patient numbers provided in the HTA report and by medical consultants during the appraisal reduces reliability of BI calculations<br>▪ Expertise by medical experts on patient numbers, medicine effectiveness and clinical pathways is considered more reliable than the findings of the HTA report limiting evidence-based decision-making<br>▪ Potential inefficiencies in committee activities given overlapping remits<br>▪ GFLAC's BI appraisal might be of lesser quality as budgets are not available to the committee<br>▪ Rejections by the committee are not communicated to the applicant resulting in appeals by industry at procurement level<br>▪ Medical experts, who are also applicants, are not always informed about the status of the application and/or whether the medicine is purchased. This results in tensions between medical consultants and patients who request updates on their treatment<br>▪ Long appraisal process demotivates medical experts to provide their expertise on new medicines

	▪ Potential conflict of interests of committee members representing both appraisal committees on the remit of the committees<br>▪ Patient perspectives are not represented in the appraisals<br>▪ Political direction to prioritize some medicines over others may result in delays of appraisals. This conflicts with EU Transparency Directive of 180 days and results in distrust of stakeholders on the process (pharmaceutical industry, medical consultants, and patients)
**Opportunities**	▪ Provision of CE evidence in HTA reports may strengthen the rational for a positive/negative recommendation by enabling the committee to evaluate the incremental benefits of new medicine(s) with the incremental costs<br>▪ Committee members may benefit from the acquired health economics expertise of the assessors
**Threats**	▪ Sales representative can influence and bias committee members and medical experts<br>▪ Lobbying by patient groups or medical expert associations can influence recommendations<br>▪ A changing landscape of political parties might result in additional medicines to be listed based on political agenda rather than population need or value for money<br>▪ European Transparency Directive of 180 days to finalize the evaluation of new medicines

	Appraisal (ACHCB)
**Strengths**	▪ The committee's expertise comprises of economist, doctors and pharmacists contributing to quality appraisals<br>▪ The inclusion of procurement representatives in the committee steers the focus on expenses<br>▪ Commitment sheets on all healthcare expenditures for the next three years are used to inform new medicine commitments and to prevent overspending<br>▪ Medical experts may be invited to the appraisal meeting for their opinion, and questions by the committee on the HTA can be posted to the assessors, both ensuring quality appraisal
**Weaknesses**	▪ Unregular appraisal meetings resulting in a backlog of assessments and delayed appraisal potentially outdating HTA reports as new competitor medicines are approved by EMA and the local authority<br>▪ Lack of time to prepare for the appraisals due to competing responsibilities limits quality evidence-based decision-making<br>▪ Committee members have limited health economic expertise to appraise CE<br>▪ Committee members apply different implicit appraisal criteria resulting in heterogenous decision-making<br>▪ Rejections by the committee are not communicated to the applicant resulting in appeals by industry at procurement level

	▪ Political direction to prioritize some medicines over others may result in delays of appraisals. This conflicts with EU Transparency Directive of 180 days and results in distrust of stakeholders on the process (pharmaceutical industry, medical consultants, and patients)<br>▪ Decisions are based on unreliable BI estimates (due to the lack of reliable epidemiological data and/or robust calculations) threatening the sustainability of the system<br>▪ Patient representatives are not part of the appraisal<br>▪ The operationalization of a budget threshold is lacking<br>▪ Recommendations of the ACHCB might be delayed in the further processes of the system (e.g., MfH based on budget availability)
**Opportunities**	▪ Growth in GDP resulting in larger budgets for new medicines<br>▪ Legitimizing CE as evaluation criterion within the required economic evaluation stipulated by legislation
**Threats**	▪ Sales representative can influence and bias committee members<br>▪ Lobbying by patient groups or medical expert associations can influence recommendations<br>▪ A changing landscape of political parties might result in additional medicines to be listed based on political agenda rather than population need or value for money<br>▪ Other responsibilities of committee members resulting in unregular appraisal meetings

	▪ Budgets for new medicines are allocated by the Ministry for Finance and are based on demand and availability of budget<br>▪ European Transparency Directive of 180 days to finalize the evaluation of new medicines
	**Decision**
**Strengths**	▪ Ear-marked budgets for specific disease areas that require considerable funds reduce impact on funds for new medicines
**Weaknesses**	▪ The allocation of budget within healthcare might lead to insufficient funds for new medicines<br>▪ The endorsement of new medicines regularly exceeds the available budget<br>▪ The remits of the supporting appraisal committees are not well stipulated in the legislation<br>▪ The allocation of budget to specific disease areas may be more political driven and less evidence-based and might displace health<br>▪ The set-up of the system (i.e., processes and procedures) are not aligned to support the EU Transparency Directive of 180 days
**Opportunities**	▪ CE evidence can improve evidence-based prioritization of disease areas and supports evidence-based funding decisions<br>▪ Partial financing of medicines through the implementation of co-payments
**Threats**	▪ The Ministry for Finance can limit the yearly budget of the MfH

	▪ Political pressure and agendas may reduce the allocation of budget to new medicines<br>▪ Lobbying by patient groups or medical expert associations can influence prioritization of disease areas and thus, availability of budget for other new medicines<br>▪ Reference prices might change considering GDP growth and Brexit increasing budgetary pressure<br>▪ High immigration rates changing the country's epidemiology require allocation of funds towards these new healthcare needs<br>▪ Free healthcare provision may not be sustainable given increases in population numbers (due to longevity and immigration), increased patient numbers with chronic diseases, and lack of co-payments. Co-payments, as contribution to the sustainability of the system, are so far unacceptable<br>▪ Entitlements are subject to change by the Ministry for the Family, Children's Rights and Social Solidarity as it decides on legally implementing additional Schedule V chronic conditions that could allow for more access to healthcare<br>▪ Mis-entitlements increase the pressure on the budget<br>▪ Unavailability of patient medication records, prescribing behaviour and therefore, stocking might lead to wastage. This impacts financial sustainability of the reimbursement system
	**Implementation: Procurement**

**Strengths**	▪ Market research to prepare for potential price negotiations and collaboration with other countries<br>▪ Clear purchasing criteria for tenders<br>▪ Use of procurement models, such as pay per cure and risk sharing agreements to facilitate access and remain within the budget<br>▪ Procurement of approved medicines is initiated only when funds are allocated by the CMO preventing unsustainable practices<br>▪ A separate committee reviews public tenders and selection reason to ensure fair competition and compliance with pricing criteria
**Weaknesses**	▪ CPSU handles the procurement of all healthcare services. The workload and competing priorities put pressure on the staff to provide medicines in a timely manner<br>▪ Procurement of medicines is delayed due to competing financial commitments. Several medicines must be purchased at the same time, but resources are lacking<br>▪ Procurement is delayed by appeals by the pharmaceutical industry on grounds of unfair competition, in cases when their medicine was not considered in the HTA report and/or rejection letters were not sent to the applicant<br>▪ Analyses are conducted to prioritize procurement of medicines limiting staff resources to procure in a timely manner<br>▪ In certain cases, the procurement unit may opt for purchasing formulations different than approved (i.e., open specifications) to save

	costs and to reduce appeals on grounds of restricted specifications by industry. Clinicians oppose this practice as it might impact patients' health<br>▪ The negotiation power of CPSU may be limited when patient numbers provided in the procurement file were wrongly estimated potentially impacting financial sustainability of the system and risking treatment availability due to either under- or overstocking<br>▪ The procurement of medicines is inflexible as changes in patient numbers after endorsement and procurement cannot be easily purchased given the yearly or two-yearly procurement cycles. Additional volumes would be purchased at a higher price or in the subsequent procurement cycle potentially impacting public health and sustainability goals<br>▪ The long periods procuring approved medicines lead to the perception by applicants that most medicines are not approved. This may result in lower submissions of applications<br>▪ The outputs of procurement are not communicated back as input to other processes (e.g., actual patient numbers and prices for following clinical pathway including that medicine)
**Opportunities**	▪ The negotiation power on prices increases through collaboration with other countries (The advent of the Valletta Technical Committee and the Brexit challenge has proved this in recent years)<br>▪ The Robotic Storage and Dispensing System in the main hospital in Malta has been launched to access data of just-in-time usage and to

	rationalise prescribing. Potentially reducing wastages but mainly assisting with data handling for new introductions
**Threats**	▪ The Minister for Finance assigns the budget for new medicines throughout the year based on demand and supply of the previous year and budget estimates for new introductions set by the Office of the CMO. If estimates in number of patients and prices, are not factual the allocated budget may not suffice<br>▪ New medicines are approved by market authorizations whilst submitted applications for the same pathway are still under appraisal resulting in additional appeals at procurement level by the pharmaceutical industry slowing down procurement and consequently access to new medicines<br>▪ Medical consultants and patients might request medicines commonly available in other EU countries or the UK that MAHs do not supply to Malta due to low margins and Brexit including export bans<br>▪ The supply of medicines is threatened by the Brexit since most of the volumes (70%) come from the UK<br>▪ The calculated MRP does not capture potential additional cost (e.g., insurance, transportation) for the transportation of medicines with certain container make-up (e.g., glass containers) and/or due to weight. This may result in less optimal procurement and thus, sustainability issues<br>▪ The fear of unavailability of medicines triggers patients into stocking pharmaceuticals in their home medicine cabinets, which may lead to wastage of the procured medicines due to expiration or non-use

	Implementation: Listing & Entitlements
**Strengths**	▪ The listing of the new medicines on the public GFL and circulating of supplementary protocols including the prescriber criteria keep stakeholders (e.g., medical consultants) informed
**Weaknesses**	▪ Patients are required to commute to central POYC for entitlements<br>▪ Inpatient pharmacies are not always aware of new medicines and, therefore, do not include them in their orders. This might result in delayed availability of new medicines as stocks are ordered 3 months in advance<br>▪ Forecasting treatment demand in acute hospitals might result in too low stocking requiring out-of-cycle procurements, which in turn disrupts the suppliers' forecasts<br>▪ The lack of a digital system that captures patient records result in high administrative paperwork prone to errors, especially when patients are transferred from home and between hospitals, and from hospital to the pharmacy<br>▪ Large volume stocks require much space at central POYC. Consequently, other medicines cannot be ordered at the same time possibly influencing availability of medicines at local pharmacies<br>▪ Lack of entitlement protocols with regards to distribution impacts both storage of medicines at in and outpatients' level resulting in patients having to collect outpatients' medicines from inpatients pharmacy or Gozitan patients having to travel to MDH to collect their medicines

**Opportunities**	▪ Changes in the treatment supply chain are expected to improve efficiency and treatment availability<br>▪ The introduction of a SMS service that reminds patients about their medicine and confirms the collection of prescribed medicines to prevent fraud
**Threats**	▪ The natural fluctuations of treatment demand limits pharmacists in predicting required stocks in advance, resulting in overstocking and consequently wastage<br>▪ The dispersion of reimbursed medicines at pharmacies that also sell medicines privately might result in fraudulent behaviour

ACHCB = Advisory Committee for Health Care Benefits; BI = Budget Impact; CE = Cost-Effectiveness; CMO = Chief Medical Officer; CPSU = Central Procurement Supply Unit; DPA = Directorate for Pharmaceutical Affairs; EMA = European Medicine Agency; EU = European Union; GDP = Gross Domestic Product; GFL = Government Formulary List; GFLAC = Government Formulary List Advisory Committee; HTA = Health Technology Assessment; MAH = Market Authorization Holder; MDH = Mater Dei Hospital; MfH = Ministry for Health; MRP = Maximum Reference Price; MRP = Maximum Reference Price; POYC = Pharmacy Of Your Choice; UK = United Kingdom; SOP = Standard Operating Procedures

Supplementary Table S2 - SWOT Exceptional Medicinal Treatments Core Processes

	**Application**
**Strengths**	▪ A new policy distinguishing three application requests (new requests, renewals and requests for previously approved indications) reduces bureaucracy tendering to medical consultants<br>▪ Short request form (1 page)
**Weaknesses**	▪ In urgent matters, the signature of the clinical chairperson, that is required on the EMT request for new indications, costs valuable time in case of absence of the chairperson<br>▪ The short request form reduces the evidence input by clinicians which then needs to be collected by the assessors
**Opportunities**	.
**Threats**	.
	**Assessment**
**Strengths**	▪ The case reports on which decisions reside upon are well conducted considering time constraints of two weeks
**Weaknesses**	.
**Opportunities**	▪ HTA reports from other HTA agencies and EUnetHTA can be utilized to inform EMT case reports
**Threats**	▪ Transferability of HTA data (e.g. economic data) from other HTA agencies and EUnetHTA could be an issue for utilization in EMT case reports
	**Appraisal & Decision**

**Strengths**	▪ Regular appraisal meetings of the EMTC (every 2-3 weeks) facilitating faster access of approved medicines<br>▪ In case of urgent requests (e.g., life-threatening), procedures are in place to handle the request within one working day
**Weaknesses**	▪ Historically, the EMT route provided a possibility to access medicines faster than when applying on the GFL. Although nowadays, requests are assessed more critically, previously approved medicines remain within the EMT route; requests are still renewed and procured<br>▪ Sometimes the committee decides for a new medicine whilst alternatives are available on the GFL, which would have been mistakenly missed<br>▪ Unclear operationalisation of reimbursement criterion 'exceptionality'<br>▪ Unclarity around budget and budgetary responsibility by EMTC; some stakeholders claimed no separate budget was available, others claimed budget was available and under the responsibility of the CMO
**Opportunities**	▪ Application of CE criterion as an opportunity for sustainability<br>▪ Application of CE criterion and threshold as a tool for balancing equitable and equal access to medicines<br>▪ Threshold suitable for orphan medicines to provide budget indication
**Threats**	.
	**Implementation: Procurement**
**Strengths**	▪ The communication between CPSU and EMTU is considered to contribute to a fast exchange of treatment specification and procurement of the EMT
**Weaknesses**	▪ Procurement does not inform subsequent processes (EMTU) on the procurement status limiting information sharing with applicants

**Opportunities**	.
**Threats**	▪ The request for many different EMT drugs reduces CPSU negotiation power due to small scales threatening, financial sustainability<br>▪ The very purpose of the EMT route limits procurement in volumes and therefore, impacts financial sustainability of the system<br>▪ The small population of Malta may have more frequently EMT needs relative to countries with larger populations<br>▪ Malta's geographic location requires some EMTs to be flown in, especially in urgent cases contributing to higher budgetary pressure
	**Implementation: Entitlements**
**Strengths**	▪ Patients receive their entitlements from central POYC via postal service and, therefore, do not have to commute to the office<br>▪ EMTs for outpatient use can be dispersed at the patient's local pharmacy allowing for fast access<br>▪ EMTs for inpatient use are sent directly to the hospital pharmacy
**Weaknesses**	.
**Opportunities**	.
**Threats**	.

CE = Cost-Effectiveness; CMO = Chief Medical Officer; CPSU = Central Procurement Supply Unit; EMT = Exceptional Medicinal Treatments; EMTC = Exceptional Medicinal Treatment Committee; EMTU = Exceptional Medicinal Treatment Unit; EU = European Union; GFL = Government Formulary List; HTA = Health Technology Assessment; POYC = Pharmacy Of Your Choice

Supplementary Table S3 Number of committee meetings and treatments appraised, per year– Government Formulary List Advisory Committee and Advisory Committee for Health Care Benefits, 2014-2022

Years	Number of GFLAC meetings	Average number of treatments appraised per GFLAC meeting	Number of ACHCB meetings	Average number of treatments appraised per ACHCB meeting
2014	9	11	1	10
2015	6	7	6	9
2016	7	5	1	4
2017	3	6	2	10
2018	5	8	3	10
2019	4	8	3	4
2020	4	6	None	None
2021	7	9	2	5
2022	7	5	7	8

(Data provided by DPA, 2022)

Supplementary Table S4 Outcomes of treatments appraised, per year – Government Formulary List Advisory Committee (GFLAC), 2014-2022

Year	Number of treatments	Approved	%	Rejected	%	Pending	%
2014	**100**	73	73%	22	22%	5	5%
2015	**40**	17	43%	9	23%	14	35%
2016	**33**	26	79%	6	18%	1	3%
2017	**17**	11	65%	5	29%	1	6%
2018	**39**	24	62%	11	28%	4	10%
2019	**30**	29	97%	1	3%	0	0%
2020	**23**	21	91%	1	4.5%	1	4.5%
2021	**66**	59	89%	2	3%	5	8%
2022	**37**	28	76%	2	5%	7	19%

Supplementary Table S5 Outcomes of treatments appraised, per year – Advisory Committee for Health Care Benefits (ACHCB), 2014-2022

Year	Number of treatments	Approved			Rejected			Pending		
		Total	*	%	Total	*	%	Total	*	%
2014	**10**	5	-	50%	1	-	10%	4	*1*	40%
2015	**54**	42	*4*	78%	6	-	11%	6	*3*	11%
2016	**4**	2	*2*	50%	0	-	0%	2	*1*	50%
2017	**21**	16	*12*	76%	4	*3*	19%	1	-	5%
2018	**30**	22	11	73%	5	4	17%	3	2	10%
2019	**12**	8	5	67%	0	0	0%	4	0	33%
2020	**0**	0	0	0%	0	0	0%	0	0	0%
2021	**10**	10	0	100%	0	0	0%	0	0	0%
2022	**57**	56	1	98%	1	0	2%	0	0	0%

*Of which oncology treatments
